# Supplementary material for: Enhanced Stability of Detergent-Free Human Native STEAP1 Protein from Neoplastic Prostate Cancer Cells upon an Innovative Isolation Procedure
Source: Int J Mol Sci. 2021 Sep 16;22(18):10012. doi: 10.3390/ijms221810012 (PMC8472055; doi:10.3390/ijms221810012)
Supplement: Supplementary file 1 [file ijms-22-10012-s001.zip › ijms-1365217-supplementary 2.pdf]

Supplementary Material

International Journal of Molecular Sciences

Enhanced Stability of Detergent-Free Human Native STEAP1 Protein from Neoplastic Prostate Cancer Cells Upon an Innovative Isolation Procedure

Jorge Barroca-Ferreira, Pedro Cruz-Vicente, Marino F. A. Santos, Sandra M. Rocha, Teresa Santos-Silva, Cláudio J. Maia and Luís A. Passarinha*

*Address correspondent to this author at UCIBIO – Applied Molecular Biosciences Unit, Departamento de Química, Faculdade de Ciências e Tecnologia, Universidade NOVA de Lisboa, 2829-516 Caparica, Portugal; Tel.: +351 275 329 069, Fax: +351 275 329 099, E-mail: lpassarinha@fcsaude.ubi.pt.

The supplementary material addresses the strategy used for the full refinement of a HIC workflow through the detailed screening of typical chromatographic parameters: buffer composition, ionic strength, and resin properties. Initially, and considering a previous work developed by our research team *(unpublished data)*, the salting-out experiments were done by combining two salts with 750 mM monosodium phosphate buffer with sodium chloride (pH 7.8) as binding buffer. The dual-salt system was reported as a promising alternative to traditional methods capable to maintain proteins solubility under low and middle ionic strengths and to increase the dynamic binding capacity without affecting the selectivity and bioactivity of the recovered fractions [26, 27]. However, both nature and concentration of the salt used in the dual-salt system should be adjusted, since it was not compatible with STEAP1 structural integrity considering that samples tended to precipitate throughout downstream processing. Alternatively, we considered using a strong salting-out ion, namely $(NH_4)_2SO_4$, which is capable of inducing a partial harsh denaturation and unfolding of the proteins [28, 29]. The polypeptide chains will expose the internal hydrophobic regions, then enhancing hydrophobic interactions [28, 29]. Previous studies developed by our research group demonstrated that $(NH_4)_2SO_4$ allowed to recover active MPs even in relatively mild elution conditions [30, 31]. Here, we tested different salt concentrations in the binding buffer as this affects the exposure of hydrophobic surface on Butyl-Sepharose ligands which will interact with hydrophobic moieties of STEAP1 to form a protein-ligand complex [25]. The ionic strength must be controlled throughout the

chromatographic process, in order to promote sample adsorption onto the column, and simultaneously to prevent the precipitation of proteins, which may lead to product denaturation, high back pressures and system cleaning difficulties [25, 32].

Considering the exposed insights, we started an exhaustive study using Butyl-Sepharose matrix. This hydrophobic resin has an intermediate length in the n-alkyl chain and acceptable levels of selectivity for the capture of proteins from the initial lysates [33, 34]. We started the initial screenings with a concentration of 1 M $(NH_4)_2SO_4$, but STEAP1 was not captured by the matrix suggesting that increasing the ionic strength could be necessary to promote the hydrophobic interactions. Indeed, a full retention of the target onto the chromatographic resin was achieved with 2 M $(NH_4)_2SO_4$, despite the elution profile denoted low selectivity since STEAP1 was detected in two distinct steps of the gradient tested (Figure 1, Peaks V and VI; SDS-PAGE – *data not shown*).

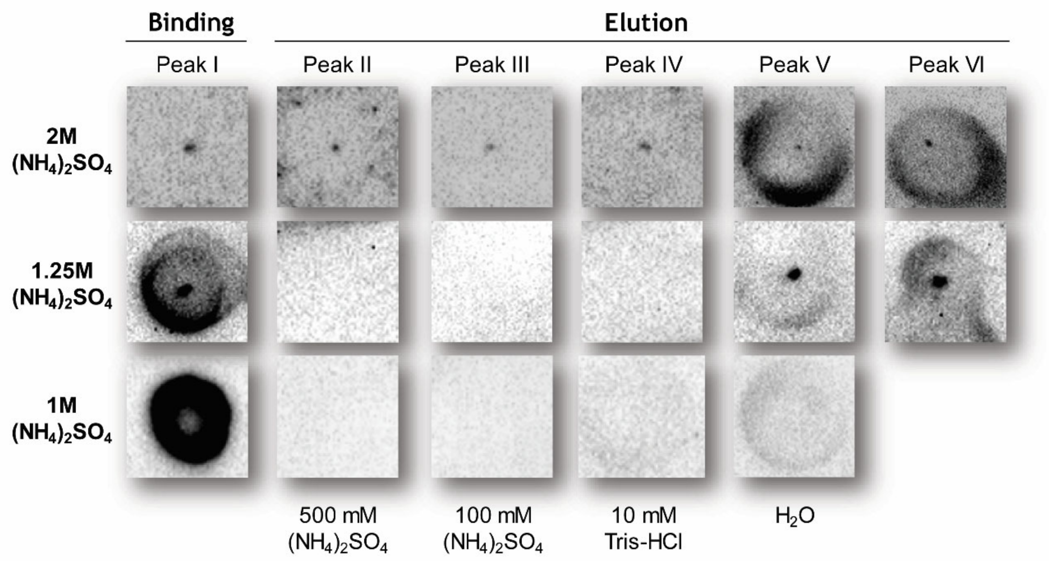

Figure S1: Dot-blot analysis of STEAP1 expression levels from purification trials on Butyl-Sepharose 4FF matrix using 1 M, 1.25 M, and 2 M $(NH_4)_2SO_4$ in binding buffer. The membranes were incubated with anti-STEAP1 mouse primary antibody overnight at 4 ºC followed by goat anti-mouse secondary antibody incubation at room temperature.

So, we further manipulated the ionic strength to 1.25 M $(NH_4)_2SO_4$. At this intermediate concentration, STEAP1 was not completely retained onto the resin. Curiously, we denoted a stronger interaction between the impurities and the column, in comparison with the target. Also, this interaction was proportionally enhanced as we increased the salt concentration of the binding buffer, as resumed by dot-blot analysis (Figure 1). This interesting feature could be important for an initial clearance of these samples. Bearing these conclusions in mind, we slightly increased the concentration of $(NH_4)_2SO_4$ to 1.375 M leading to considerable amounts of a fraction of highly

pure STEAP1 in the flowthrough (Figure 2, Peak I), with an estimate concentration of 50 μM upon pre-purification trials from the LNCaP cells crude extract.

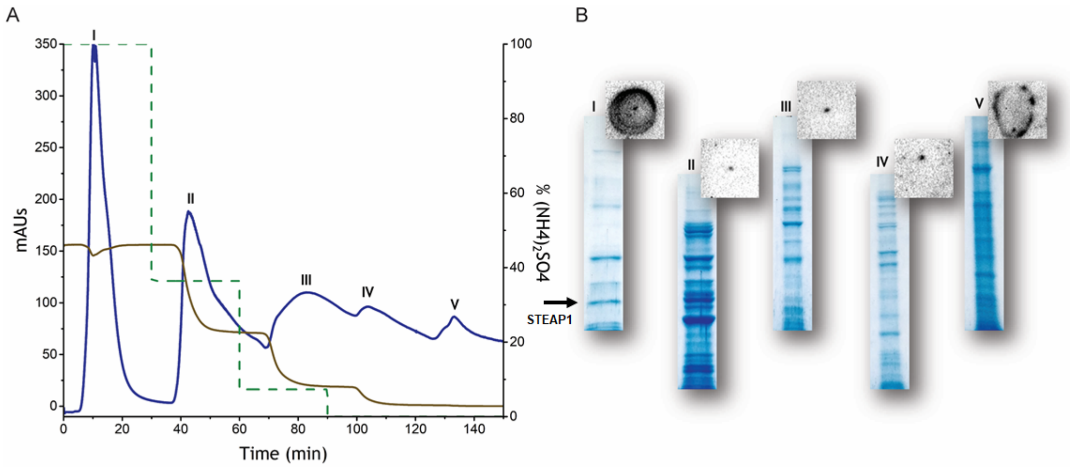

Figure S2: A) Chromatographic profile of STEAP1 purification on Butyl-Sepharose 4 FF resin with optimized conditions. Blue line represents absorbance at 280 nm. Adsorption performed at 1.375 M $(NH_4)_2SO_4$ in 10 mM Tris-HCl buffer at pH 7.8 (1.0 mL min^{-1}). Desorption was done at 500 mM and 100 mM $(NH_4)_2SO_4$, 10 mM Tris-HCl and a final step with H_2O (1.0 mL min^{-1}), in stepwise gradient. B) SDS-PAGE and dot-blot analysis depicted for each peak. The gel was stained with Coomasie brilliant blue and the membranes were incubated with anti-STEAP1 mouse primary antibody overnight at 4 ºC followed by goat anti-mouse secondary antibody incubation at room temperature. STEAP1 is marked with a black arrow.

At this moment, we considered crucial to test another matrix, as Octyl-Sepharose, with distinct ligand properties. This resin is often classified as a strong hydrophobic gel as a consequence of an increase in the n-alkyl chain length, although it has a lower density of ligands, compromising the adsorption specificity [25, 32]. These characteristics encouraged us to assess not only if the previously defined strategy could be replicated but also to refine the purity of STEAP1 recovered extract. Following a parallel strategy, 1 M $(NH_4)_2SO_4$ was used in the initial trials, with very similar results to those obtained for Butyl-Sepharose matrix, by which we decided to use the ideal salt concentration previously established for this resin. Interestingly, upon 1.375 M $(NH_4)_2SO_4$, although completely retained onto the column, STEAP1 was fractionated in a non-selective manner, which forced us to decrease the ionic strength to 1.1 M $(NH_4)_2SO_4$, as summed by dot-blot analysis (Figure 3).

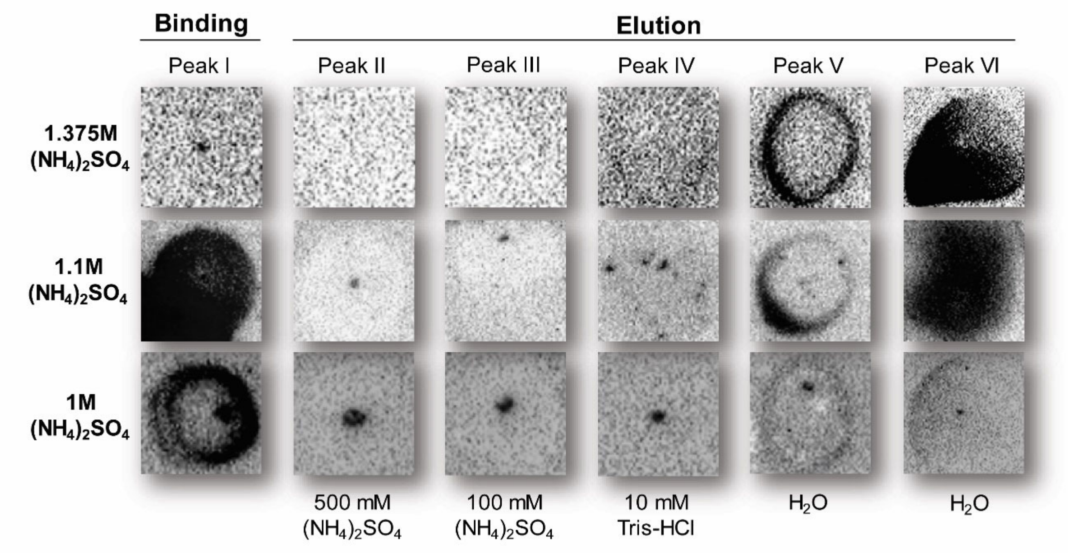

Figure S3: Dot-blot analysis of STEAP1 expression levels from purification trials on Octyl-Sepharose 4FF matrix using 1 M, 1.1 M, and 1.375 M $(NH_4)_2SO_4$ in binding buffer. The membranes were incubated with anti-STEAP1 mouse primary antibody overnight at 4 ºC followed by goat anti-mouse secondary antibody incubation at room temperature.

Despite STEAP1 was not captured by the matrix, the number of interfering compounds that co-elute with the protein of interest was still increased *(*SDS-PAGE - *data not shown)*. Once again, we adjusted the concentration of $(NH_4)_2SO_4$ to 1.2 M, whereas a considerable amount of STEAP1 was successfully isolated with a residual number of impurities (Figure 4, Peak I). Nevertheless, when compared to Butyl-Sepharose, the amount of target protein that was lost as a consequence of its co-elution with water was higher (Figure 4, Peak VI).

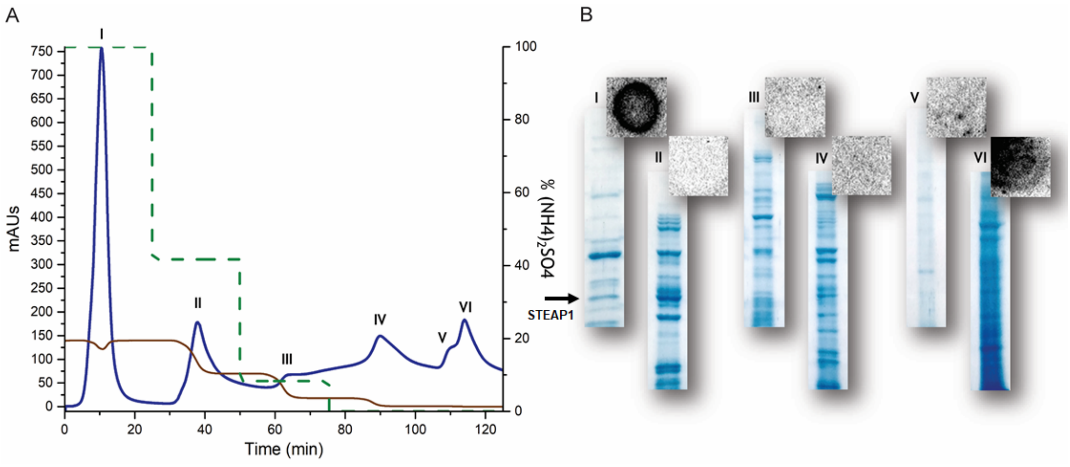

Figure S4: A) Chromatographic profile of STEAP1 purification on Octyl-Sepharose 4 FF resin with optimized conditions. Blue line represents absorbance at 280 nm. Adsorption performed at 1.2 M $(NH_4)_2SO_4$ in 10 mM Tris-HCl buffer at pH 7.8 (1.0 mL min^{-1}). Desorption was done at 500 mM and 100 mM $(NH_4)_2SO_4$, 10 mM Tris-HCl and a final step with H_2O (1.0 mL min^{-1}), in stepwise gradient. B) SDS-PAGE and dot-blot analysis depicted for each peak. The gel was stained with

Coomasie brilliant blue and the membranes were incubated with anti-STEAP1 mouse primary antibody overnight at 4 ºC followed by goat anti-mouse secondary antibody incubation at room temperature. STEAP1 is marked with a black arrow.

References

25. Tomaz, C. T., Hydrophobic interaction chromatography. In *Liquid Chromatography*, Elsevier: 2017; pp 171-190.
26. Nunes, V.; Bonifácio, M.; Queiroz, J.; Passarinha, L., Assessment of COMT isolation by HIC using a dual salt system and low temperature. *Biomedical Chromatography* **2010,** 24, (8), 858-862.
27. Sousa, A.; Passarinha, L.; Rodrigues, L.; Teixeira, J.; Mendonça, A.; Queiroz, J., Separation of different forms of proteose peptone 3 by hydrophobic interaction chromatography with a dual salt system. *Biomedical Chromatography* **2008,** 22, (5), 447-449.
28. Hofmeister, F., Zur lehre von der wirkung der salze. *Archiv für experimentelle Pathologie und Pharmakologie* **1888,** 25, (1), 1-30.
29. Porath, J., Salt-promoted adsorption chromatography. *Journal of Chromatography A* **1990,** 510, 47-48.
30. Passarinha, L.; Bonifácio, M.; Soares-da-Silva, P.; Queiroz, J., A new approach on the purification of recombinant human soluble catechol-O-methyltransferase from an Escherichia coli extract using hydrophobic interaction chromatography. *Journal of Chromatography A* **2008,** 1177, (2), 287-296.
31. Passarinha, L.; Bonifacio, M.; Queiroz, J., Comparative study on the interaction of recombinant human soluble catechol-O-methyltransferase on some hydrophobic adsorbents. *Biomedical Chromatography* **2007,** 21, (4), 430-438.
32. O'Connor, B. F.; Cummins, P. M., Hydrophobic Interaction Chromatography. In *Protein Chromatography*, Springer: 2017; pp 355-363.
33. Hahn, R.; Deinhofer, K.; Machold, C.; Jungbauer, A., Hydrophobic interaction chromatography of proteins: II. Binding capacity, recovery and mass transfer properties. *Journal of Chromatography B* **2003,** 790, (1-2), 99-114.
34. Machold, C.; Deinhofer, K.; Hahn, R.; Jungbauer, A., Hydrophobic interaction chromatography of proteins: I. Comparison of selectivity. *Journal of Chromatography A* **2002,** 972, (1), 3-19.
